# Health Psychology Consultation in the Inpatient Medical Setting

**DOI:** 10.5195/jmla.2020.904

**Published:** 2020-04-01

**Authors:** Elizabeth Connor

**Affiliations:** Professor of Education, The Citadel, Charleston, SC, elizabeth.connor@citadel.edu

Clinical psychologist Susan M. Labott is board certified in clinical health psychology, has extensive experience directing inpatient and outpatient health psychology services, and serves as a professor of clinical psychology in the University of Illinois Hospital and Health Sciences System. Labott’s practical and well-organized work is intended for clinical psychologists who are not experienced or familiar with providing consultation to inpatients hospitalized for other medical conditions. She explains that “psychological practice in the inpatient medical setting is different in many ways from both outpatient clinical work and inpatient work in a psychiatric unit” (p. 3).

The book is organized into five main sections: I, “The Inpatient Setting and Consultation Models”; II, “The Inpatient Evaluation”; III, “Psychological Issues in the Inpatient Setting”; IV, “Special Issues in the Hospital Setting”; and V, “Ethics and Professional Issues.” Section II, “The Inpatient Evaluation,” provides practical information for preparing, interviewing, and documenting psychology consultations. Practical information includes sample admission notes, progress notes, discharge summaries, and consultation reports. Section III, “Psychological Issues in the Inpatient Setting,” covers adjustment, anxiety, depression, delirium, cognitive changes, and substance abuse. Section IV, “Special Issues in the Hospital Setting,” details issues such as decision-making abilities, noncompliance, pain, and end of life. Labott’s keen interest and expertise in ethics are evident in Section V, “Ethics and Professional Issues.” The professional issues discussed in this section include training, billing, productivity, and professional identity.

The book includes two appendixes (“Common Medical Abbreviations” and “Common Laboratory Tests and Their Interpretation”); a twenty-seven-page list of references; and a fifteen-page index. *Health Psychology Consultation in the Inpatient Medical Setting* is suitable and recommended for any collection that focuses on clinical psychology or interprofessional consultation.

***Elizabeth Connor, MLS, MEd, AHIP***, elizabeth.connor@citadel.edu, Professor of Education, The Citadel, Charleston, SC

